# Large piezoelectric strain with ultra-low strain hysteresis in highly *c*-axis oriented Pb(Zr_0.52_Ti_0.48_)O_3_ films with columnar growth on amorphous glass substrates

**DOI:** 10.1038/s41598-017-13425-w

**Published:** 2017-10-10

**Authors:** Minh D. Nguyen, Evert P. Houwman, Guus Rijnders

**Affiliations:** 1 Inorganic Materials Science, MESA+ Institute for Nanotechnology, University of Twente, P.O. Box 217, 7500AE Enschede, The Netherlands; 2 Solmates B.V., Drienerlolaan 5, 7522NB Enschede, The Netherlands; 3 International Training Institute for Materials Science, Hanoi University of Science and Technology, Dai Co Viet 1, Hanoi 10000 Vietnam

## Abstract

Thin films of PbZr_0_._52_Ti_0_._48_O_3_ (PZT) with largely detached columnar grains, deposited by pulsed laser deposition (PLD) on amorphous glass substrates covered with Ca_2_Nb_3_O_10_ nanosheets as growth template and using LaNiO_3_ electrode layers, are shown to exhibit very high unipolar piezoelectric strain and ultra-low strain hysteresis. The observed increase of the piezoelectric coefficient with increasing film thickness is attributed to the reduction of clamping, because of the increasingly less dense columnar microstructure (more separation between the grains) with across the film thickness. A very large piezoelectric coefficient (490 pm/V) and a high piezoelectric strain (~0.9%) are obtained in 4-µm-thick film under an applied electric field of 200 kV/cm, which is several times larger than in usual PZT ceramics. Further very low strain hysteresis (*H*≈2–4%) is observed in 4 to 5 µm thick films. These belong to the best values demonstrated so far in piezoelectric films. Fatigue testing shows that the piezoelectric properties are stable up to 10^10^ cycles. The growth of high quality PZT films with very large strain and piezoelectric coefficients, very low hysteresis and with long-term stability on a technologically important substrate as glass is of great significance for the development of practical piezo driven microelectromechanical actuator systems.

## Introduction

Microelectromechanical systems (MEMS) actuators driven by piezoelectric materials such as lead zirconate titanate with the morphotropic phase boundary, PbZr_0.52_Ti_0.48_O_3_ (PZT), composition, have received wide attention because they can potentially outperform other MEMS actuators, because of the remarkably high ferroelectric polarization and piezoelectric coefficients of PZT^1,2^. For most MEMS device applications, such as vibration energy harvesters, micropumps, microcantilever-based mass sensors and micromachined ultrasonic transducers for medical and sonar applications, the (effective) transverse piezoelectric coefficient (*e*_31f_ or *d*_31f_) of the film is the most important factor to be considered. For specific applications however, such as nanometer-position control systems based on piezoelectric actuators for the control of optical cavities or short wavelength mirrors^3^, a large (effective) longitudinal piezoelectric coefficient (*d*_33f_) is required to obtain a large piezoelectric deformation in the PZT thin film capacitors.

The high piezoelectric response in ceramic PZT is due not only to the deformation of the crystal unit cells (intrinsic effect) but also due to the motion of domain walls, polarization switching and the motion of phase boundaries (extrinsic effect)^4^. Domain wall motion and domain switching contributes in a significant way to the increase of piezoelectricity, but it is only useful these processes are reversible. However, polarization switching is in an unipolar driven actuator device an irreversible process and the associated significant piezoelectric enhancement is useless^5^. There is evidence that nonlinear behaviour is related to the domain switching in the piezoelectric materials^6,7^. Under an applied electric field the spontaneous polarization of domains may be reoriented, which is commonly called ferroelectric of ferroelastic domain switching. The nonlinearity (or strain hysteresis) that appears in piezoelectric material is also ascribed to the domain switching related irreversible polarization and piezoelectric strain.

For actuators used in practice, the electric field is applied tom a component along its poling axis (3-direction). The resulting electric field-induced piezoelectric strain (*S*_3_) can be described as:

1 $${S}_{3}={s}_{33}{{\rm{\sigma }}}_{3}+{S}_{3}^{^{\prime} }({E}_{3})+{d}_{33}^{\ast }{E}_{3}$$

where, s_33_σ_3_ represents the mechanical elastic strain, S′_3_ and *d* ^*^_33_ *E*_3_ are the domain switching-related irreversible strain and the contribution of the reversible inverse piezoelectric effect, respectively. A large-signal electric field leads to ferroelastic domain switching in the piezoelectric materials. Due to the change of strain, the material parameters in Eq. (1) are no longer constant, but depend on the history of the applied electric field. As a result, the actual response of piezoelectric materials will display significant hysteresis and nonlinearity.

Piezoelectric materials with (strongly) enhanced longitudinal piezoelectric displacement (or piezoelectric strain) were presented. However most studies reporting on large strain are limited to bulk ceramics of piezoelectric materials due to the difficulty of growing thin films with similar piezoelectric response as in ceramic form. Lead-based piezoelectric ceramics, such as the soft PZT (PZT-5H) ceramics have a maximum strain (*S*_max_) of 0.175%, corresponding to a normalized piezoelectric coefficient ${d}_{33}^{\ast }\,(\equiv {S}_{max}/{E}_{max})$ of 730 pm/V and a relative strain hysteresis ($H={({S}_{forw}-{S}_{ret})}_{{E}_{max}/2}/{S}_{max}$ with ${S}_{forw}({S}_{ret})$ the strain on the rising (falling) branch of the hysteresis loop at half the maximum applied field *E*_*max*_. At maximum field the strain is *S*_*max*_) of 12% for *E*_max_ = 24 kV/cm. A lower strain (*S* = 0.105%, equivalent to ${d}_{33}^{\ast }$ = 250 pm/V) and a lower *H* value of 5% were obtained in the hard PZT (PZT-8) ceramics, for *E*_max_ = 42 kV/cm^8^.

Many studies have been performed to find lead-free piezoelectric ceramics as an alternative for the PZT materials. Yao *et al*. reported that the large piezoelectric coefficient (*d*_33_) of 280 pm/V, and a strain of 0.28% (under an *E*_max_ of 56 kV/cm), and a high normalized ${d}_{33}^{\ast }$ coefficient of 833 pm/V (at low *E*_max_ of 18 kV/cm) in (K,Na,Li)(Nb,Ta,Sb)O_3_ ceramics^9^. Similar high values (*S* = 0.23% at *E*_max_ = 50 kV/cm) were obtained in the (lead containing) Pb(Yb_0.5_Nb_0.5_)O_3_-PbHfO_3_-PbTiO_3_ composition^10^. Recently very high strains have been observed in (Fe-Sb)-codoped (Bi_0.5_Na_0.5_)_1−x_Ba_*x*_Ti_0.98_O_3_ (BNT–BT) ceramics (*S* = 0.57%, ${d}_{33}^{\ast }$ = 713 pm/V at *E*_max_ = 80 kV/cm)^11^ and in NaNbO_3_-doped Bi_0.5_Na_0.5_TiO_3_–Bi_0.5_K_0.5_TiO_3_ (BNT–BKT) ceramics (*S* = 0.445%, ${d}_{33}^{\ast }$ = 810 pm/V at *E*_max_ = 55 kV/cm)^12^. However, the strain hysteresis (*H*) values of these ceramics were very high, such as *H*≈70% for the (Fe-Sb)-codoped BNT–BT system^11^ and *H*≈60% for the NaNbO_3_-doped BNT–BKT system^12^. These results indicate that high values of strain in piezoelectric ceramics are often accompanied by large strain hysteresis, which limits the useful application in piezoelectric actuators.

Piezo-driven actuators have recently been used in adaptive optics, deformable mirrors, camera modules, as well as in high-resolution positioning stages with the range of motion from nano- to micro-meters^13–15^. For the integration of piezoelectric materials in such systems, in many cases the piezoelectric materials should be prepared in thin-film form on glass substrates. Although the lead-free ceramics have high piezoelectric responses, there is great difficulty in growing these materials in thin film form with similar piezoelectric response as in ceramic form. Therefore piezoelectric MEMS devices based on PZT are nowadays still widely used in both research and development and in commercial products. In a previous paper, we demonstrated that the piezoelectric coefficient (*d*_33f_) of PZT films deposited on Pt/Ti/SiO_2_/Si by PLD, can be enhanced strongly by using vertically oriented columnar growth^16^. A high *d*_33f_ value of 408 pm/V was found in 4 µm thick PZT films, which corresponds fairly well with the theoretical result of Cao *et al*. (525 pm/V for *x*_Ti_ = 0.48)^17^. This value is much larger than the theoretical value for PZT single-crystal and single-domain from Haun *et al*. (327 pm/V)^18^ and also larger than typical experimental values found for bulk PZT ceramics (*d*_33_ = 223 pm/V)^19^. We also investigated the effect of microstructure and orientation on the piezoelectric properties of such PZT films. Further we demonstrated a high *d*_33f_ of 356 pm/V in columnar grown (001)-oriented 2-µm-thick PZT films on amorphous glass substrates^20^.

In this work, we describe the fabrication of (001)-oriented PbZr_0.52_Ti_0.48_O_3_ (PZT) thin films on Ca_2_Nb_3_O_10_ nanosheets (CNO*ns*) coated amorphous glass substrates, using conductive oxide LaNiO_3_ (LNO) layers as top and bottom electrodes. We achieve very high piezoelectric strain and low strain hysteresis as a result of the vertically oriented columnar growth technique. We investigate in detail the influence of film thickness on the microstructure and piezoelectric response. Extremely large strain response of 0.9% was obtained in 4-µm-thick PZT films with ${d}_{33}^{\ast }=450$ pm/V, together with a low strain hysteresis of 4% for *E*_*max*_ = 200 kV/cm. A very large ${d}_{33}^{\ast }$ value of 690 pm/V was obtained in this film for *E*_*max*_ = 55 kV/cm (or an applied voltage of 22 V), resulting in a displacement of 15 nm. In addition, to evaluate the long-term reliability, fatigue measurements of unipolar strain/displacement were also performed.

## Results and Discussion

By varying the PZT film thickness changes of the microstructure over the thickness were obtained in films grown on LaNiO_3_ (LNO) buffered CNO*ns*/glass substrates. We observe notable effects of these differences in the ferroelectric and piezoelectric properties of these PZT film capacitors that we relate to changes in substrate clamping.

In order to investigate the effect of the film thickness on the structures and electrical properties, several PZT films with thicknesses ranging from 1 to 5 µm were prepared using PLD. Fig. S2a shows the XRD patterns of PZT films with different thicknesses deposited on LNO buffered CNO*ns*/glass substrates at a laser repetition frequency of 50 Hz. Clearly all the films have crystallized in a pure perovskite phase with a (001)-preferred orientation and no evidence of secondary phase formation, such as the pyrochlore phase, was detected. As the film thickness increases, the peak intensity of the (001) plane increases as well whereas the peak intensity of the (110) plane decreases, indicating that (110) growth mainly occurs at the bottom of the film and is suppressed when the film growth continues. The crystalline quality was further determined from the rocking curve widths of the PZT(002) reflections as shown in Fig. S2b. With increasing film thickness the FWHM value of the rocking curve increases, indicating that the average grain tilt increases, but maintaining the (001) growth orientation in the grains.

**Figure 1:**
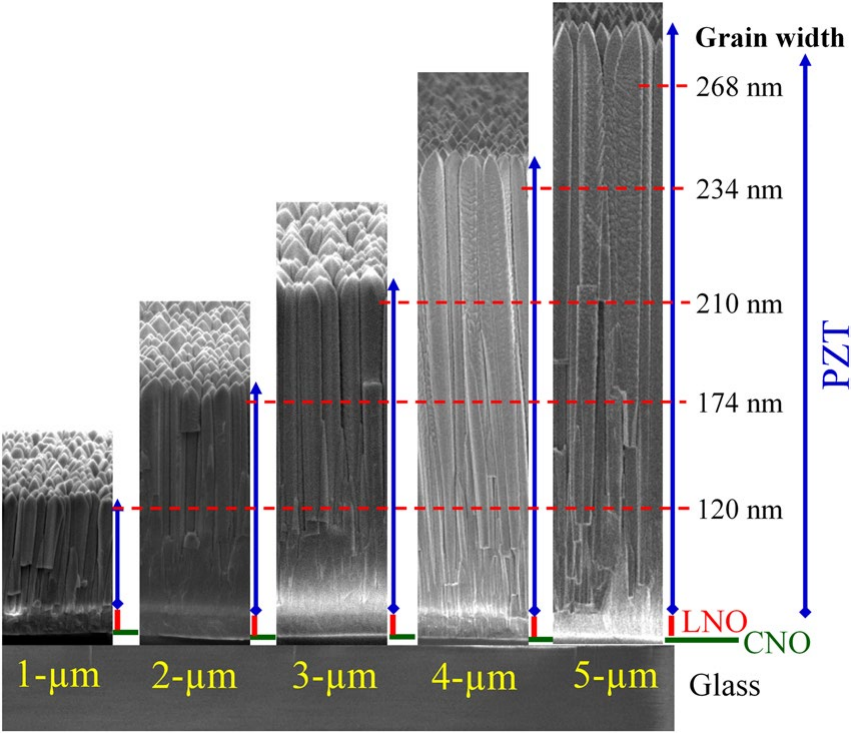
Cross-sectional SEM images of PZT films with various thicknesses deposited on LNO/CNO*ns*/glass.

AFM and SEM micrographs of the surfaces and cross-sections of the PZT films deposited on CNO*ns*/glass are shown in Fig. S3 and Fig. 1, respectively. From the AFM surface micrographs it is seen that the grain diameter and the distances between the grains become larger with film thickness. AFM line scans spectra in Fig. S4 show an increase in the surface height fluctuations of the films with increasing thickness from about 70 nm for the 1 µm film to 190 nm for the 5 µm film. The root-mean square surface roughness (*R*_rms_) increases from 23 to 56 nm. The SEM cross-sectional images show that the columnar grains extend through the complete thickness of the PZT films. The average width of the grains at the top of the layers increases from about 120 to 268 nm as the film thickness changes from 1 to 5 µm (see Table 1). Previously it was also shown that for the used deposition conditions with increasing thickness the columnar grains become increasingly detached from each other, reducing the effect of film clamping by the substrate^16^.

**Table 1:** Properties of PZT films as a function of film thickness.

Film thickness (µm)	Rocking curve FWHM of ω-scan of PZT(002) (°)	*R*_*rms*_ (nm)	Column diameter *d*_*col*_ (nm) ^a^	*P*_*r*_ (µC/cm^2^)	*E*_*c*_ (kV/cm)	*d*_*33f*_^*b*^ (pm/V)	${d}_{33}^{\ast }$ ^c^ (pm/V)
1.0	0.55	23.2	120	22.0	37.4	227	265
2.0	0.67	32.1	174	28.1	35.0	356	390
3.0	0.96	39.3	210	30.0	30.3	460	458
4.0	1.16	42.7	234	30.0	30.2	490	450
5.0	1.34	56.0	268	27.4	25.7	454	430

^a^at the top of the layer; ^*b*^determined at *E* = 0 kV/cm from *d*_*33f*_ *−E* hysteresis loops up to 200 kV/cm at 1 kHz scan frequency; ^*c*^determined from unipolar *S-E* loops up to 200 kV/cm at 10 Hz scan frequency.

**Figure 2:**
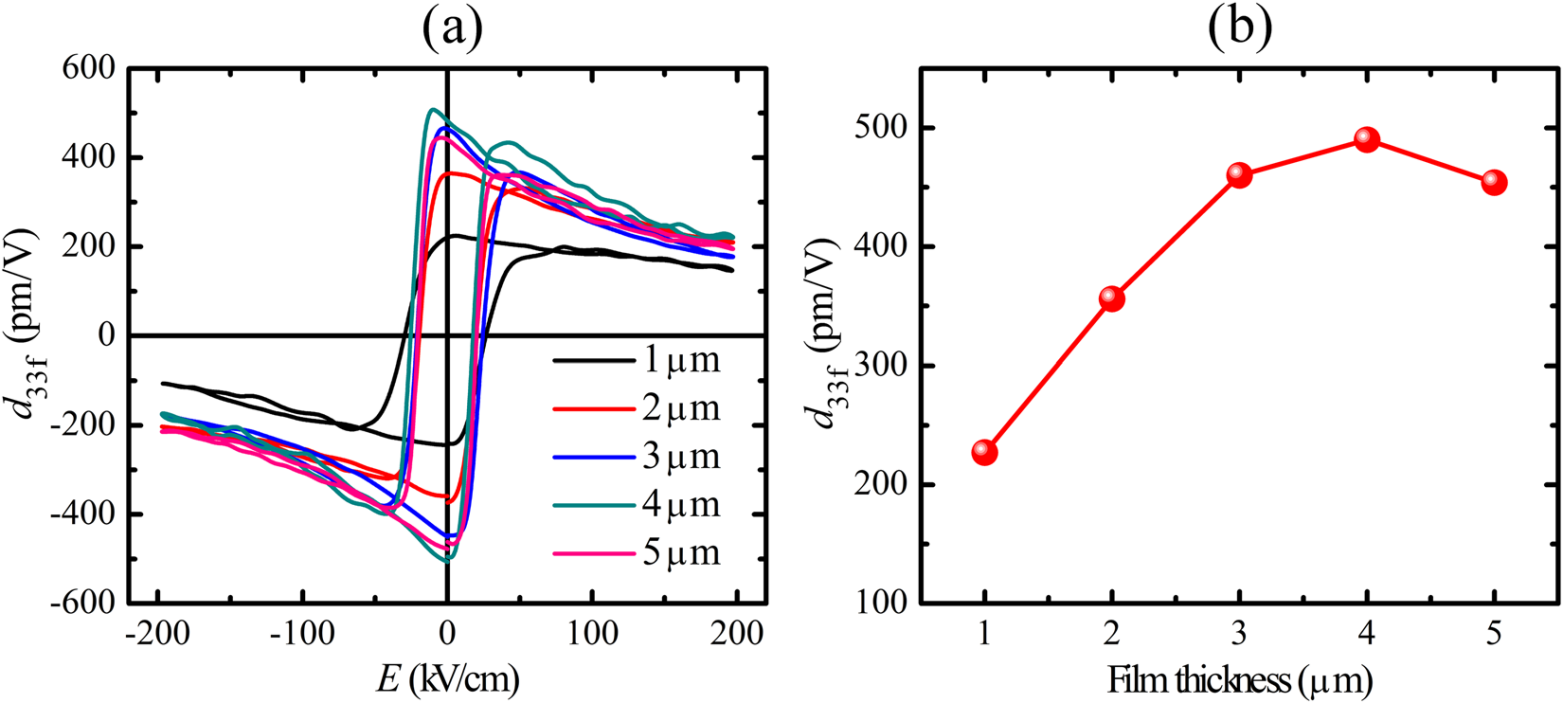
(**a**) Small-signal *d*_33f_–*E* loops and **(b)** thickness dependence of *d*_33f_, of PZT films with varying thicknesses deposited on LNO/CNO*ns*/glass.

Ferroelectric and piezoelectric properties of the PZT films have been investigated as a function of thickness. Fig. S5a shows the *P-E* hysteresis loops. The remanent polarization (*P*_r_) value increases with film thickness and reaches a maximum value of about 30.0 µC/cm^2^ at a thickness of 3 µm, while the coercive field (*E*_c_) decreases continuously with increasing film thickness (Fig. S5b). The electric-field dependence of the small-signal effective piezoelectric coefficient (*d*_33f_–*E*) for the different film thicknesses is shown in Fig. 2a. The zero field *d*_33f_ values increase from 227 to 490 pm/V as the film thickness increases from 1 to 4 µm, and then slightly decreases for the 5 µm film (Fig. 2b). The Fig. 2a further indicates that the *d*_33f_ value strongly decreases with increasing electric field. In this case the polarization vectors are largely aligned parallel to the applied field and only the intrinsic piezoelectric effect contributes to *d*_33f_. Therefore, the *d*_33f_ values peak in the low electric field region, where extrinsic contributions to the piezoelectric effect are largest and gradually decreases at higher electric fields where wall motion does not contribute to the piezoelectric effect any more.

The effect of film thickness on the ferroelectric and piezoelectric properties can be explained by the contribution of domain structure changes related to the change in substrate clamping. Ferroelectric and piezoelectric properties are generally considered to originate from both intrinsic and extrinsic contributions as was demonstrated by Kim *et al*.^21^ and Xu *et al*.^22^. The intrinsic contribution originates from the response of a single polarization domain, whereas extrinsic contributions originates from the motion of the domain walls and phase boundaries^23^. The bottom of the films are tensile clamped due to thermal expansion coefficient difference between the film (*TEC* *≈* 5 ppm/K) and the substrate (*TEC* *≈* 0 ppm/K), causing some polarization rotation towards the film plane, but at larger thickness the grains are detached and the grains in the film are increasingly strain relaxed and the polarization rotates more in the out of plane direction. Therefore, an enhanced *P*_r_ value is obtained for less tensile stress, which is here the case with increasing film thickness^24^. Similarly the polarization domains in the films are less clamped due to the reduction of substrate constraints, so that reverse domain nucleation becomes easier and then the *P*_r_ value increases while the coercive field decreases with increasing film thickness. Further the increased effective *d*_33f_ coefficient with increasing film thickness is caused by a reduction of the clamping and the associated enhancement of the domain wall motion and switching in the increasingly freestanding grains^25^. In the previous study^16^, we have also discussed on the effect of film thickness on the microstructure and properties of PZT films grown on Pt/Ti/SiO_2_/Si (Pt/Si) substrates, in which the grains in the films can be considered to be polydomain single crystals. With increasing thickness the grains become less connected with neighbouring grains (as can be seen in Fig. S4 where the surface height fluctuations of the films increase with increasing thickness) and the effect of clamping on domain wall motion, polarization rotation and crystal unit cell deformation is reduced. Table 1 indicates that the values of *P*_r_ and piezoelectric coefficient (*d*_33f_ or ${d}_{33}^{\ast }$) are reasonably saturated at the film thickness of 3–4 µm. However, this saturated thickness value is not constant and depends on the many factors such as the type of ferroelectric films, electrodes and substrates. Kim *et al*. indicated that the epitaxial PZT films grown on SrTiO_3_ and SrTiO_3_/Si substrates have the saturated thickness of about 2 µm^25^ and similar to the case of polycrystalline PZT films on Pt/Si^26^, while the maximum values of ferroelectric and piezoelectric properties were obtained at the film thickness of about 1 µm in the epitaxial PZT films grown on YSZ/Si substrates^24^. However, the maximum value of *P*_r_ was obtained at quite thinner film (330 nm) in the BiFeO_3_ thin films deposited on SrRuO_3_ buffered Pt/Si substrates^27^.

A high value of the effective piezoelectric coefficient *d*_33f_ is the important parameter for e.g., sensors or ultrasonic devices; while the normalized strain piezoelectric coefficient (${d}_{33}^{\ast }$) is the key parameter that matters in actuator systems. The normalized strain ${d}_{33}^{\ast }$ value is calculated as:

2 $${d}_{33}^{\ast }=S({E}_{max})/{E}_{max}$$

**Figure 3:**
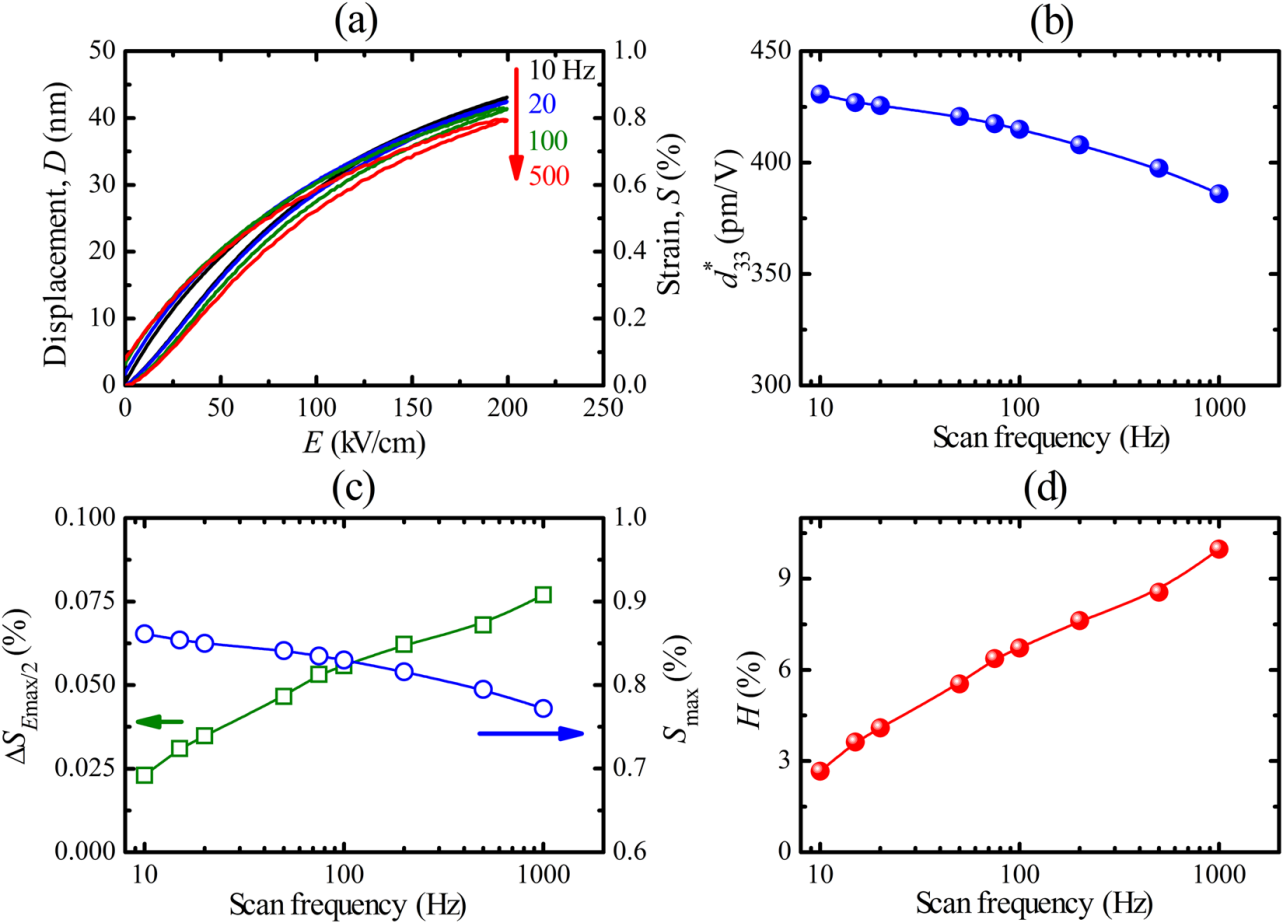
(**a**) Unipolar piezoelectric displacement (*D*–*E*) and strain versus *E*-field (*S*–*E*) loops as a function of scan frequency for 5 µm PZT film. Scan frequency dependence of **(b)** normalized ${d}_{33}^{\ast }$ at *E*_max_, **(c)** fraction of the unipolar strain at *E*_max_/2 (Δ*S*_*E*max/2_) and strain at *E*_max_ (*S*_max_) and **(d)** strain hysteresis (*H*), with *E*_max_ = 200 kV/cm.

**Figure 4:**
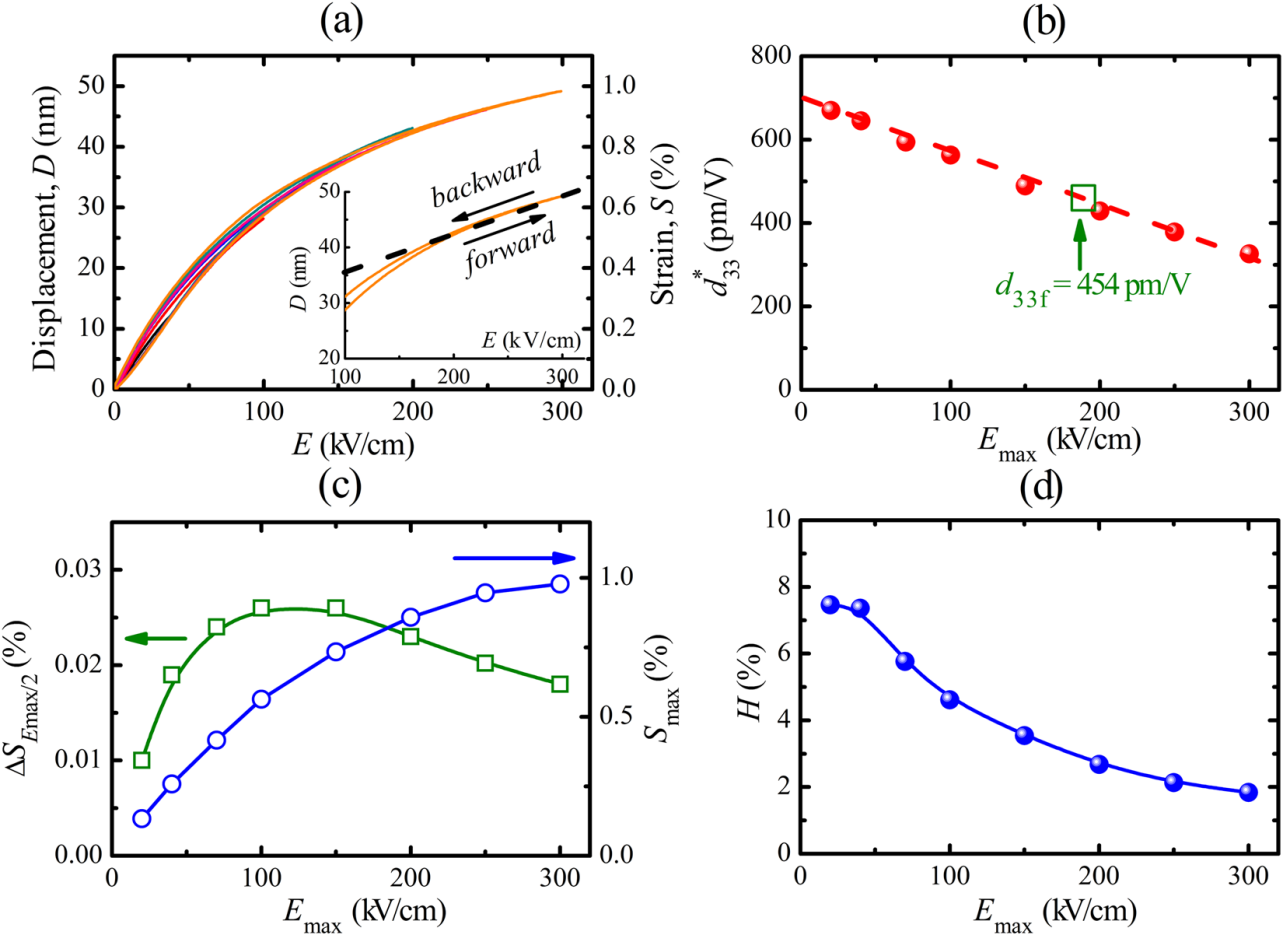
(**a**) Unipolar piezoelectric displacement (*D*–*E*) and strain versus *E*-field (*S*–*E*) loops for 5-µm-thick PZT film. *E*–field dependence of **(b)** normalized ${d}_{33}^{\ast }$ at *E*_max_, **(c)** fraction of the unipolar strain at *E*_max_/2 (Δ*S*_*E*max/2_) and strain at *E*_max_ (*S*_max_), and **(d)** strain hysteresis (*H*).

In order to evaluate the piezoelectric strain and strain hysteresis responses for practical applications in actuators, the dependence on the measurement (scan) frequency and the maximum applied electric field is investigated. In this study, the unipolar strain and strain hysteresis behaviour of a 5-µm-thick PZT film was evaluated as a function of scan frequency from 10 to 1000 Hz (at an electric field of 200 kV/cm; see Fig. 3a) and as a function of applied electric field up to 300 kV/cm at 10 Hz (Fig. 4a).

Figure 3a–d show the unipolar piezoelectric displacement (*D*–*E*), the unipolar strain (*S*–*E, S* = 100% × *D*/*t*, where *t* is the PZT film thickness) curves, the maximum strain (*S*(*E*_max_) or *S*_max_), the normalized ${d}_{33}^{\ast }$ values and the relative strain hysteresis (*H*), that is related to the piezoelectric loss, of the 5 µm film as a function of scan frequency. Both *S*_max_ (Fig. 3c) and ${d}_{33}^{\ast }$ (Fig. 3b) values slightly decline when the frequency increases, whereas *H* values (Fig. 3d) significantly increase with increasing frequency. *S*_max_ and ${d}_{33}^{\ast }$ reach maximum values (0.86% and 431 pm/V) and low *H* value (2.7%) is obtained at the scan frequency of 10 Hz. It is known that the domain wall motion and domain wall switching are a time dependent process^28–30^, thus one can expect that with increasing driving frequencies the *H* value is increased, and *S*_max_ and ${d}_{33}^{\ast }$ values are slightly decreased.

The piezoelectric strain (*S*–*E*) curves and normalized ${d}_{33}^{\ast }$ values of the 5-µm-thick PZT film as a function of applied electric field (scan frequency: 10 Hz) are presented in Fig. 4a and b. The maximum strain values gradually increase with increasing applied electric field reaching a maximum value of about 1% at *E*_max_ = 300 kV/cm (or *V*_max_ = 150 V), as shown in Fig. 4c. Simultaneously *H* drops continuously with increasing *E*_max_ (Fig. 4d). This huge *S*_max_ value (~1%) with ultra-low strain hysteresis (*H*≈1.8%) belongs to the best values for piezoelectric films. The *S*_max_ value used in this study is much higher than that of for example PZT4 bulk ceramics (*S* = 0.35% at *E*_max_ = 20 kV/cm)^31^. The inset of Fig. 4a shows that the forward and backward displacements are nearly overlapping in the 150–300 kV/cm range which is of great advantage for real application in actuators.

The ${S}_{max}\mbox{--}{E}_{max}$ curve is linear up to about *E*_*max*_ = 15 kV/cm and above this value the slope decreases and the curve tends to saturate (Fig. 4c). Therefore a large ${d}_{33}^{\ast }$ value of about 670 pm/V is obtained at a low applied electric field of 20 kV/cm, as indicated in Fig. 4b, but ${d}_{33}^{\ast }$ decreases approximately linearly with increasing *E*_*max*_. It is also interesting to note that the ${d}_{33}^{\ast }$ value at low applied electric field (<150 kV/cm) is much larger than the small-signal piezoelectric coefficient value (at zero field). In both measurement methods (small-signal measurement for *d*_33f_ and large-signal measurement for ${d}_{33}^{\ast }$) one expects that the intrinsic piezoelectric effect is approximately the same for both the small-signal *d*_33f_–*E* and the large-signal unipolar strain *S*–*E*. On the other hand, the extrinsic contribution is sensitive to the mode of external excitation. For the *d*_33f_–*E* measurement, a low external *ac* voltage of 400 mV was applied, a value well below the coercive field, thus there is negligible contribution from domain wall depinning and subsequent motion. There is still an extrinsic contribution from domain wall oscillation. The piezoelectric unipolar strain was measured with a large *ac* amplitude well-above the coercive field that makes domain wall depinning possible and significant domain wall motion. Therefore one may expect that a higher extrinsic contribution from domain wall motion to the strain is the origin of the larger ${d}_{33}^{\ast }$ value as compared to *d*_33f_ at an electric field below 150 kV/cm. As discussed above at higher fields there is increasingly less domain wall motion, since all polarization becomes aligned leading to the decrease in ${d}_{33}^{\ast }$ and *d*_33f_(*E*).

The effect of electric-field induced domain wall motion and switching can also be seen in the strain hysteresis (Fig. 4d). The strain hysteresis (*H*) is high at the low electric fields, reflecting the large contribution from the pinning of domain wall motion, but with increasing field this effect decreases, since the contribution of domain wall motion to the total strain decreases.

**Figure 5:**
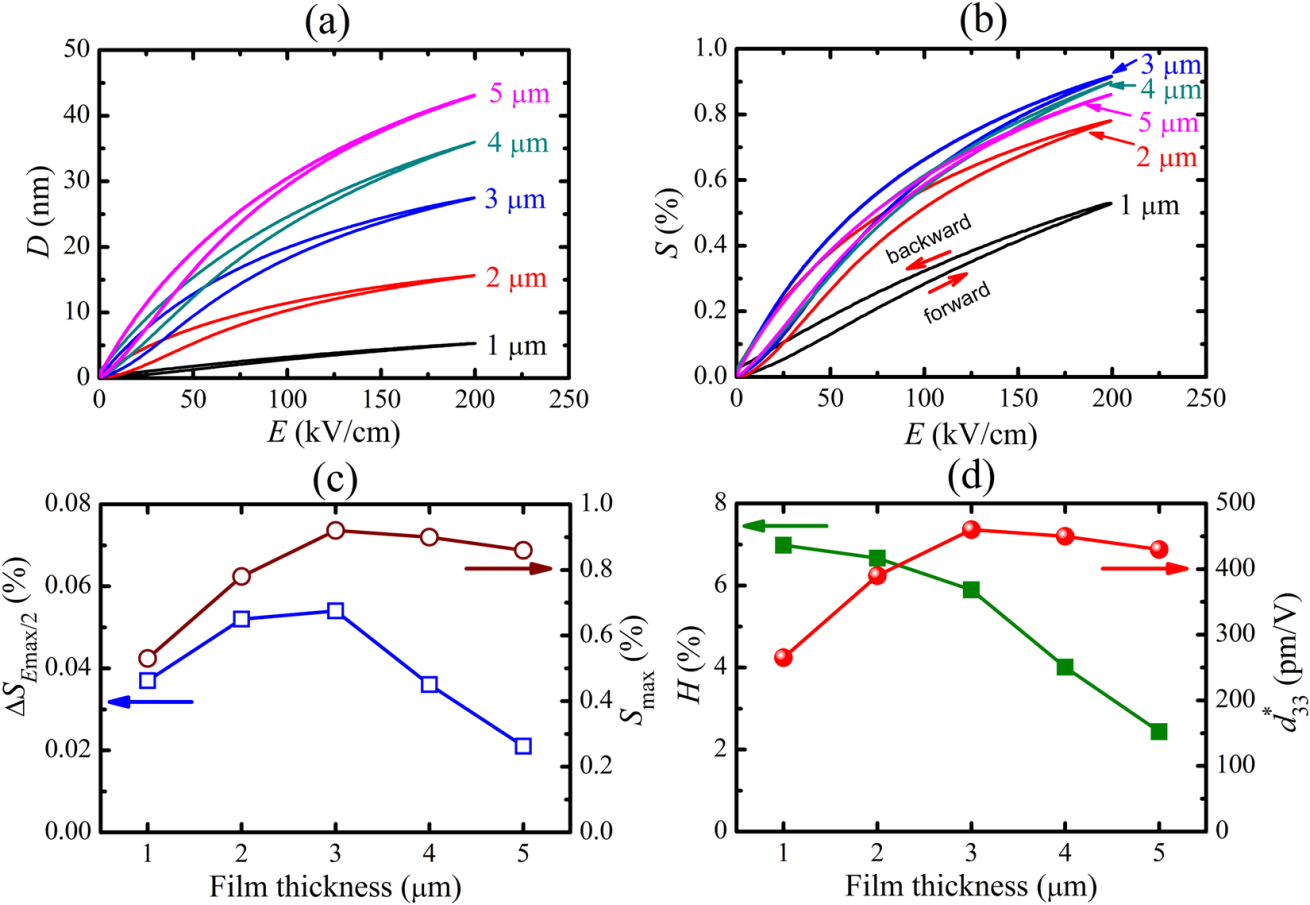
**(a)** Unipolar piezoelectric displacement versus *E*–field (*D*–*E*) and **(b)** unipolar strain versus field (*S*–*E*) loops of PZT films with different thicknesses. Thickness dependence of **(c)** *S*_max_ and fraction of the unipolar strain at *E*_max_/2 (Δ*S*_*E*max/2_), and **(d)** large-field ${d}_{33}^{\ast }$ and relative strain hysteresis (*H*). The measurements were done at a scan frequency of 10 Hz.

In order to investigate the effect of film thickness on the hysteresis in the piezoelectric displacement and strain, the unipolar piezoelectric displacements of PZT films in the range of 1–5 µm thick were measured with a maximum applied electric field of 200 kV/cm and at 10 Hz scan frequency. Slim piezoelectric displacement (*D*–*E*) curves are observed for all films (Fig. 5a). The large enhancement of the maximum displacement from 5.3 nm for the 1-µm-thick film to 43.0 nm for 5-µm-thick film implies that the displacement increases more than linear with the thickness. Of course the required driving voltage also increases from 20 to 100 V. Figure 5b shows the unipolar strain curves measured up to 200 kV/cm. Note that the largest strains are obtained for the films with thickness in the range 3–4 µm. The maximum strain and ${d}_{33}^{\ast }$ values of PZT films are presented in Fig. 5c–d. It is seen that the *S*_max_ and ${d}_{33}^{\ast }$ values increase with increasing film thickness and reach the maximum values of 0.92% and 460 pm/V for the film thickness of 3 µm. On further increasing thickness *S*_max_ and ${d}_{33}^{\ast }$ values decrease.

Figure 5d also shows the relative strain hysteresis (*H*) of the films with different thicknesses. *H* decreases continuously with increasing film thickness reaching a very low *H* value of 2.4% in the 5-µm-thick film (at *E*_*max*_ = 200 kV/cm). The reduced *H* is partly due to the reduced opening Δ*S* (Fig. 5c) of the strain loops for thicknesses larger than 2–3 µm, which suggests reduced domain wall pinning, and partly due to the increasing value of *S*_*max*_ with increasing thickness.

**Figure 6:**
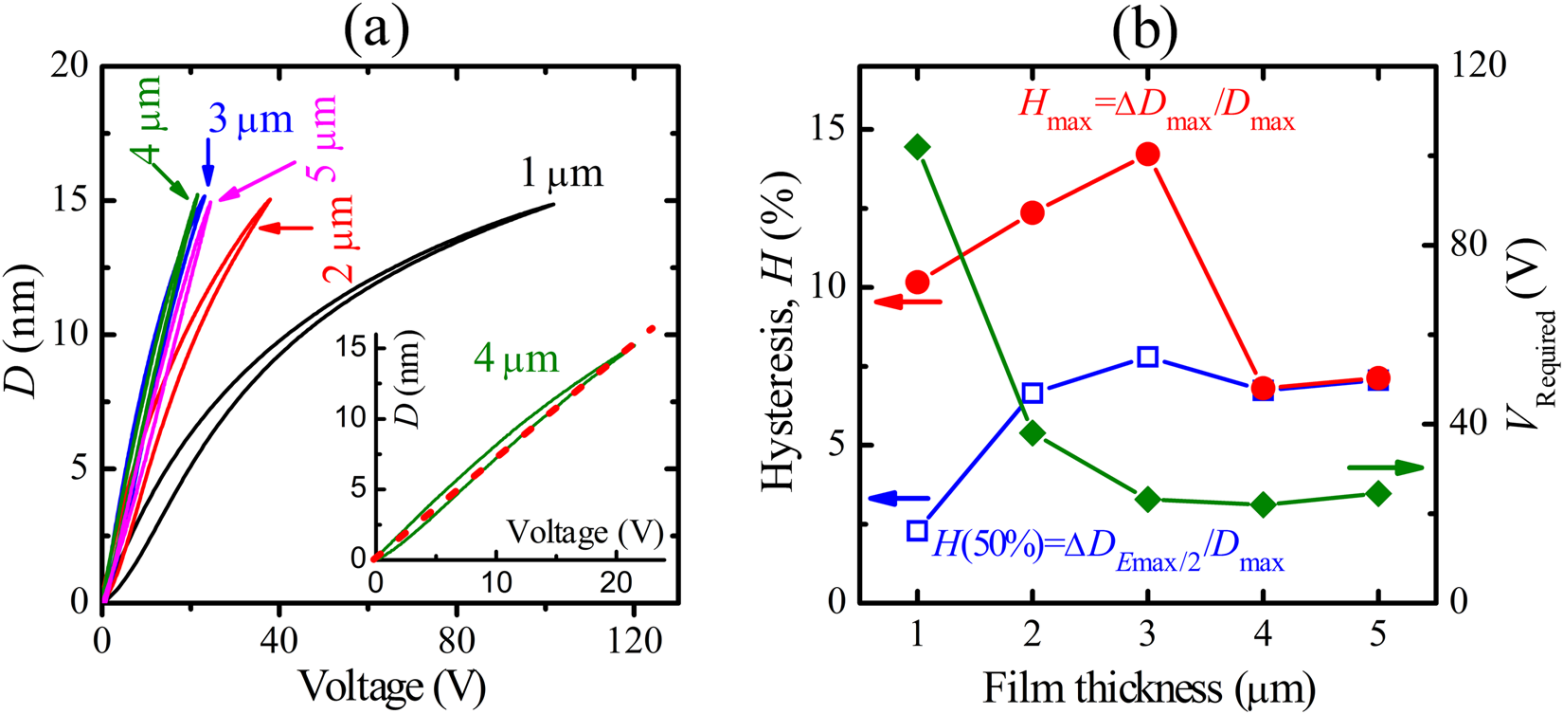
**(a)** Unipolar piezoelectric displacement (*D*–*V*) loops, and **(b)** Strain hysteresis defined at Δ*D*_Emax/2_ and Δ*D*_max_ and required applied voltage (*V*_required_) for the about 15-nm displacement of film capacitors. The measurements were done at a scan frequency of 10 Hz.

The above results demonstrate that for the same applied field, an enhanced piezoelectric displacement and low strain hysteresis are obtained in the thicker PZT films. However for some specific applications a specific maximum piezoelectric displacement is required. Figure 6a shows the unipolar piezoelectric displacement curves of PZT films with the same maximum displacement of 15 nm, by adjusting the maximum applied voltage (or electric field). The required voltages are about 102, 38, 23.2, 22 and 24.5 V for the films with the thickness of 1, 2, 3, 4 and 5 µm, respectively (${d}_{33}^{\ast }$ values from 0 V up to this voltage are respectively 146, 396, 653, 690 and 620 pm/V), as shown in Fig. 6b. The lowest applied voltage is needed for the 3–4 µm thick films. This means that low voltage driven actuators can be fabricated that achieve large piezoelectric displacements. This may open up good prospects for the application of such piezo-electric films. The films show the low train hysteresis of about 7–7.5%, except for the 1-µm-thick film with *H* value of 2.3% due to the high applied voltage in the measurement.

Moreover, Fig. 6a also indicates that the piezoelectric strain value as high as 0.375% under low electric field of 55 kV/cm is obtained in the 4-µm-thick PZT film. This value is comparable with the superior strain in lead-free materials, such as NaNbO_3_-doped Bi_0.5_Na_0.5_TiO_3_–Bi_0.5_K_0.5_TiO_3_ (0.445%)^12^, 0.965K_0.45_Na_0.55_Nb_0.98_Sb_0.02_O_3_–0.035Bi_0.5_Na_0.5_Zr_0.85_Hf_0.15_O_3_ (0.325%)^32^, but in the bulk ceramic form. Further the piezoelectric coefficient ${d}_{33}^{\ast }$ of 690 pm/V in the 4-µm-thick PZT film is also equivalent to that in the lead-free (Bi_0.5_Na_0.5_)TiO_3_–BaTiO_3_ (BNT-based) and (Ba,Ca)(Zr,Ti)O_3_ ceramics^33^. This also confirms the applicability of our PZT films for piezoelectric MEMS actuator devices.

**Figure 7:**
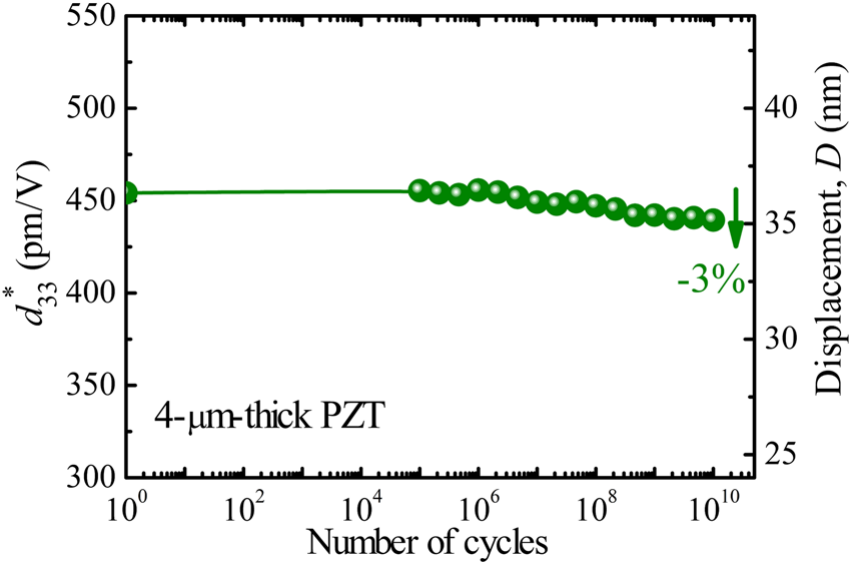
Piezoelectric displacement and large-signal ${d}_{33}^{\ast }$ value of 4-µm-thick PZT film as a function of working cycles, defined from the unipolar *D-E* loops measured at 200 kV/cm and 10 Hz. The fatigue testing was performed by applying a bipolar triangular field of 100 kV/cm and at 100 kHz.

In addition to the large piezoelectric coefficient, high piezoelectric strain and low strain hysteresis, long-term stability of these properties under working conditions is essential for the application of such films in practical devices. Figure 7 shows the piezoelectric displacement (and ${d}_{33}^{\ast }$) of the 4-µm-thick PZT film up to 10^10^ bipolar cycles (100 kV/cm at 100 kHz). Hardly any fatigue is present this piezoelectric film, suggesting that these films have excellent potential for demanding high cycle applications such as in MEMS actuators.

## Conclusions

We have shown that the microstructure of PZT films changes over the thickness of thick films. With increasing thickness the columnar grains become more separated and consequently less clamped by each other and the substrate. This causes a change in the piezoelectric and ferroelectric properties. The piezoelectric coefficients are significantly higher for the thicker films. A large *d*_33f_ of 490 pm/V, and a high strain of 0.9% (${d}_{33}^{\ast }\,$= 450 pm/V) with a low strain hysteresis of 4% were observed in 4-µm-thick PZT film measured at 200 kV/cm. For a displacement of 15 nm the applied voltage is only 22 V (corresponding to *E* = 55 kV/cm), which amounts to a very large ${d}_{33}^{\ast }$ value of 690 pm/V over the 0–55 kV/cm voltage range. The joint effects of vertically (001)-oriented columnar growth and thickness contribution (the film-electrode interfacial layer and substrate constrains effects are reduced with the thicker film) were identified to be the origin of the superior piezoelectric performance in this film. The combination of large piezoelectric strain and low strain hysteresis makes these films very suitable for piezoelectric actuator applications that require large displacements. Moreover, excellent fatigue resistance was observed in the films up to 10^10^ bipolar cycles. This finding demonstrates that these materials have excellent potential for demanding high cycle applications such as MEMS actuators.

## Methods

Pb(Zr_0.52_Ti_0.48_)O_3_ (PZT) films were grown on 200-nm-thick LaNiO_3_ (LNO) electrodes which in turn were deposited on CNO*ns* coated glass substrates using pulsed laser deposition (PLD) with a KrF excimer laser source (Lambda Physik, 248 nm wavelength). In this study, Ca_2_Nb_3_O_10_ (CNO*ns*) nanosheets were fabricated on glass substrates by exfoliation of layered protonated calcium niobate, HCa_2_Nb_3_O_10_•1.5H_2_O, followed by the Langmuir-Blodgett (LB) deposition method. The details of the flux synthesized, layered precursor KCa_2_Nb_3_O_10_ and its protonation process can be found in previous papers^34,35^. An AFM image of a monolayer CNO nanosheet on glass is shown in Fig. S1. The deposition conditions for the PZT films were: laser repetition rate 50 Hz, energy density 2.5 J/cm^2^, oxygen pressure 0.1 mbar and a substrate temperature of 600 °C. For the LNO electrodes the deposition conditions were 4 Hz, 2.5 J/cm^2^, 0.1 mbar O_2_ and 600 °C. All layers were deposited successively without breaking the vacuum. After deposition the films were cooled down to room temperature in a 1 bar oxygen atmosphere at a ramp rate of 8 ^o^C/min.

The crystal structure of the thin films was analyzed by X-ray diffraction *θ*–2*θ* scans (XRD, Philips X’Pert X-ray diffractometer). The microstructure was investigated using atomic force microscopy (AFM: Bruker Dimension Icon). For electrical measurements, 300 × 300 µm^2^ capacitors were patterned with a standard photolithography process and structured by argon-beam etching of the top-electrodes and wet-etching (*HF*-*HCl* solution) of the PZT films. The ferroelectric hysteresis (*P-E*) loops were measured with the ferroelectric mode of the aixACCT TF-2000 Analyzer, using a triangular *ac*-electric field of ±200 kV/cm at 1 kHz scanning frequency. The zero-field longitudinal piezoelectric coefficient (*d*_33f_) of the piezoelectric thin-film capacitors was defined from the hysteresis loop of the piezoelectric coefficient (*d*_33f_–*E*), measured by a double-beam laser interferometer (aixDBLI) method with a *dc* driving field between ±200 kV/cm on which an *ac* voltage of 400 mV (corresponding to 4 kV/cm for a 1-µm-thick film, down to 0.8 kV/cm for a 5-µm-thick film) and 1 kHz was superimposed (small-signal measurement). For the piezoelectric strain (*S*–*E*) measurement, an unipolar triangular-shaped *ac* electric field was applied to the film with 200 or 300 kV/cm amplitude at 10 Hz and the piezoelectric displacement was traced at the same time (large-signal measurement). The piezoelectric cycling fatigue measurements were performed with a bipolar switching pulse of 100 kV/cm pulse height at 100 kHz repetition frequency.

## Electronic supplementary material

Supplementary Information
