# Supplementary material for: Large piezoelectric strain with ultra-low strain hysteresis in highly c-axis oriented Pb(Zr0.52Ti0.48)O3 films with columnar growth on amorphous glass substrates
Source: Sci Rep. 2017 Oct 10;7:12915. doi: 10.1038/s41598-017-13425-w (PMC5635137; doi:10.1038/s41598-017-13425-w)
Supplement: Supplementary file 1 — Supplementary Information [file 41598_2017_13425_MOESM1_ESM.pdf]

Supplementary Information

Large piezoelectric strain with ultra-low hysteresis in highly *c*-axis oriented $Pb(Zr_{0.52}Ti_{0.48})O_3$ films with columnar growth on amorphous glass substrates

Minh D. Nguyen[1,2,3,*], Evert P. Houwman[1], and Guus Rijnders[1]

[1]Inorganic Materials Science, MESA+ Institute for Nanotechnology, University of Twente, P.O. Box 217, 7500AE Enschede, The Netherlands

[2]Solmates B.V., Drienerlolaan 5, 7522NB Enschede, The Netherlands

[3]International Training Institute for Materials Science, Hanoi University of Science and Technology, Dai Co Viet 1, Hanoi 10000, Vietnam

* E-mail: d.m.nguyen@utwente.nl

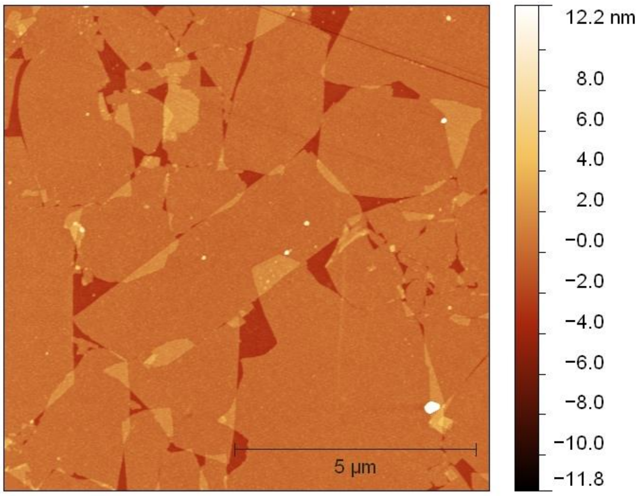

Figure S1. AFM image of a monolayer $Ca_2Nb_3O_{10}$ (CNO*ns*) nanosheet on an amorphous glass substrate. The thickness of a monolayer of CNO*ns* is about 2.6 nm.

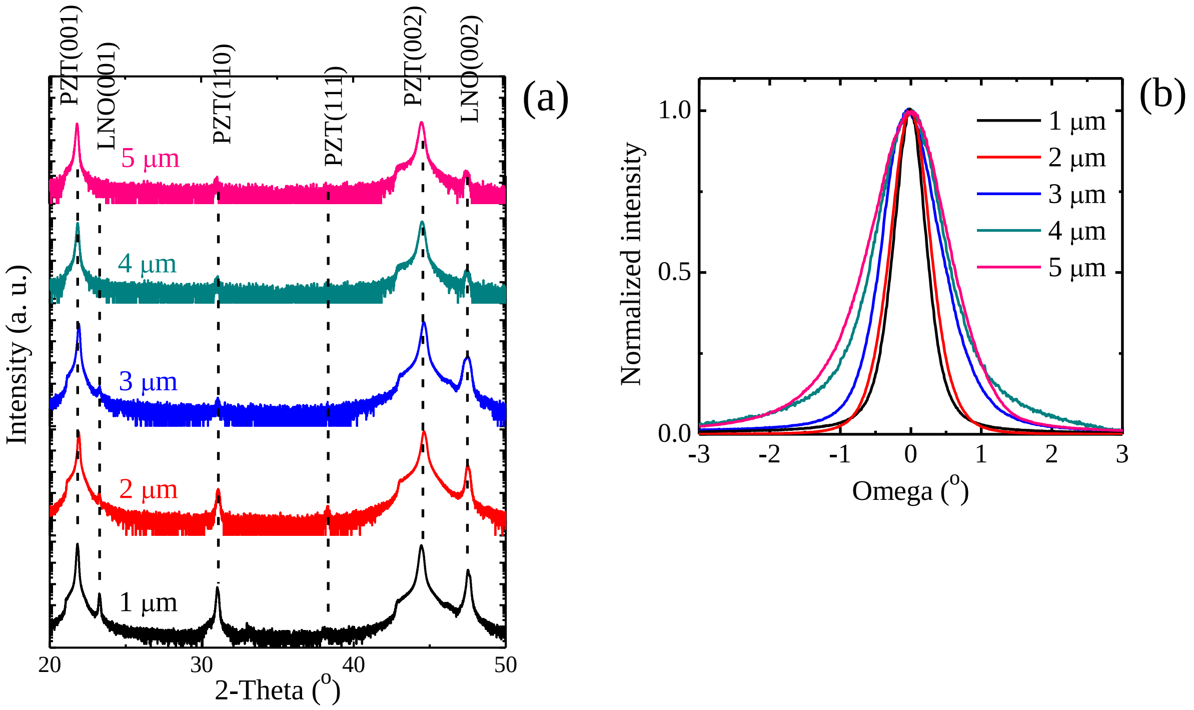

Figure S2. XRD patterns of (a) θ–2θ scan and (b) rocking curve, of PZT films grown on LNO/CNO*ns*/glass as a function of film thickness.

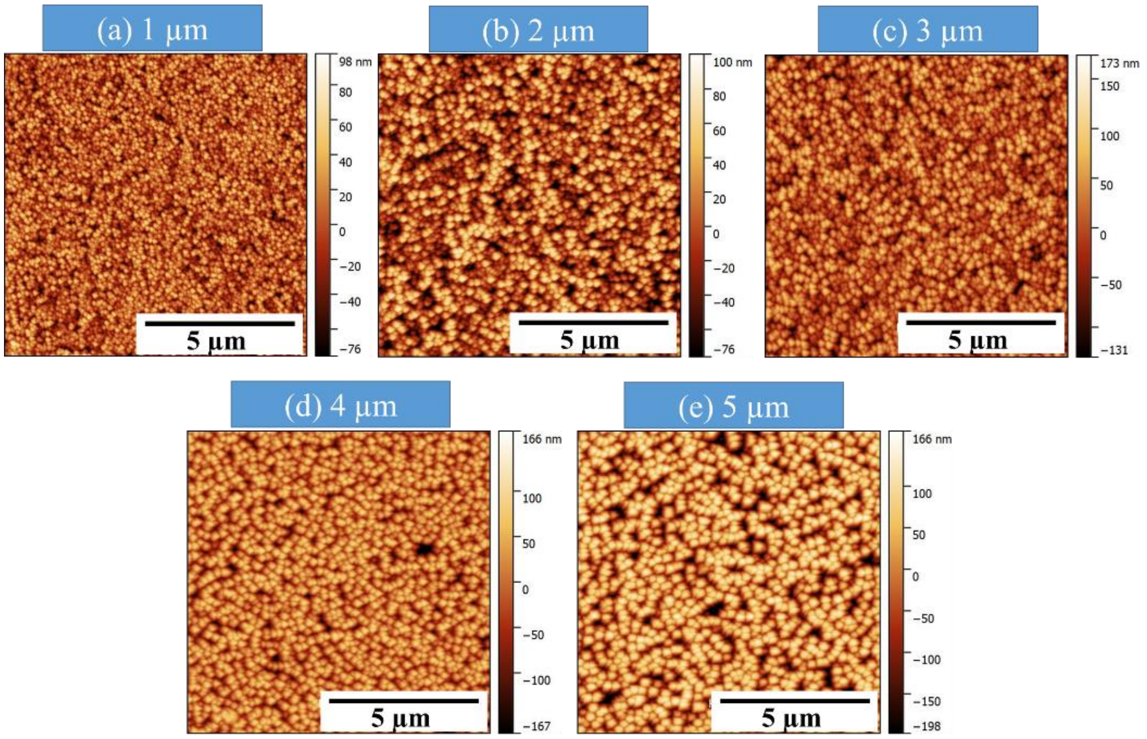

Figure S3. AFM images of PZT films with various thicknesses deposited on LNO/CNO*ns*/glass.

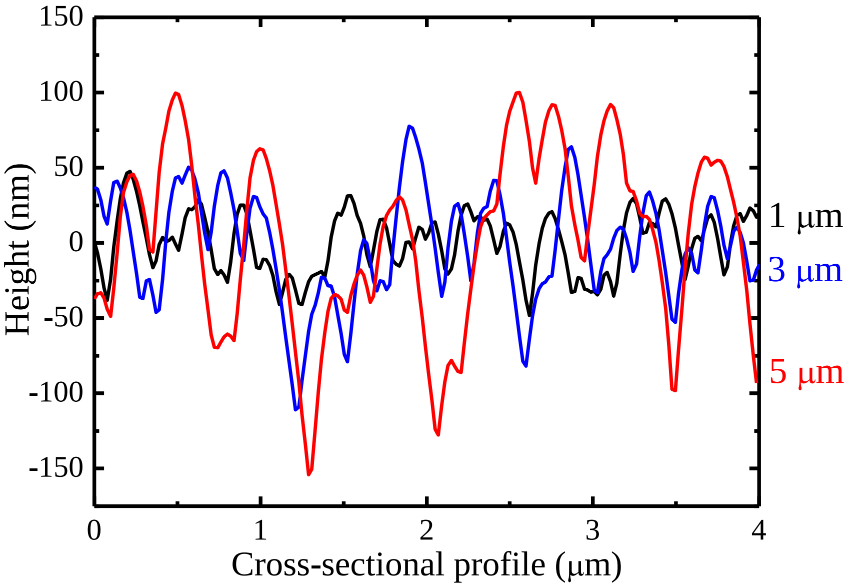

Figure S4. Height profiles of the PZT films grown on CNO*ns*/glass substrates extracted from digitized AFM images for different thicknesses.

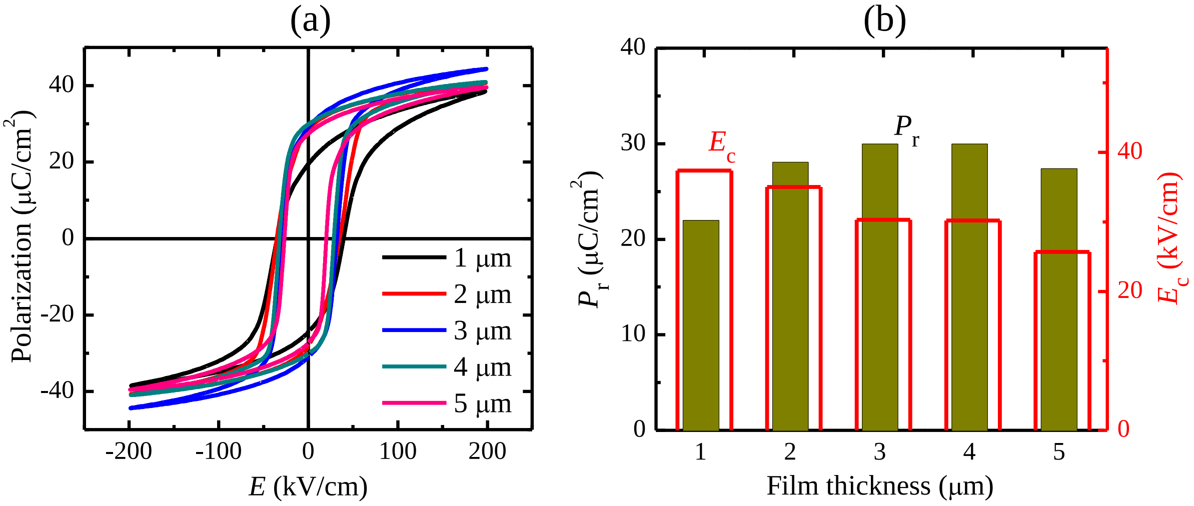

Figure S5. (a) Ferroelectric polarization hysteresis (*P*-*E*) loops and (b) remanent polarization (P_r) and coercive field (E_c) values, of PZT films with various thicknesses deposited on LNO/CNO*ns*/glass.

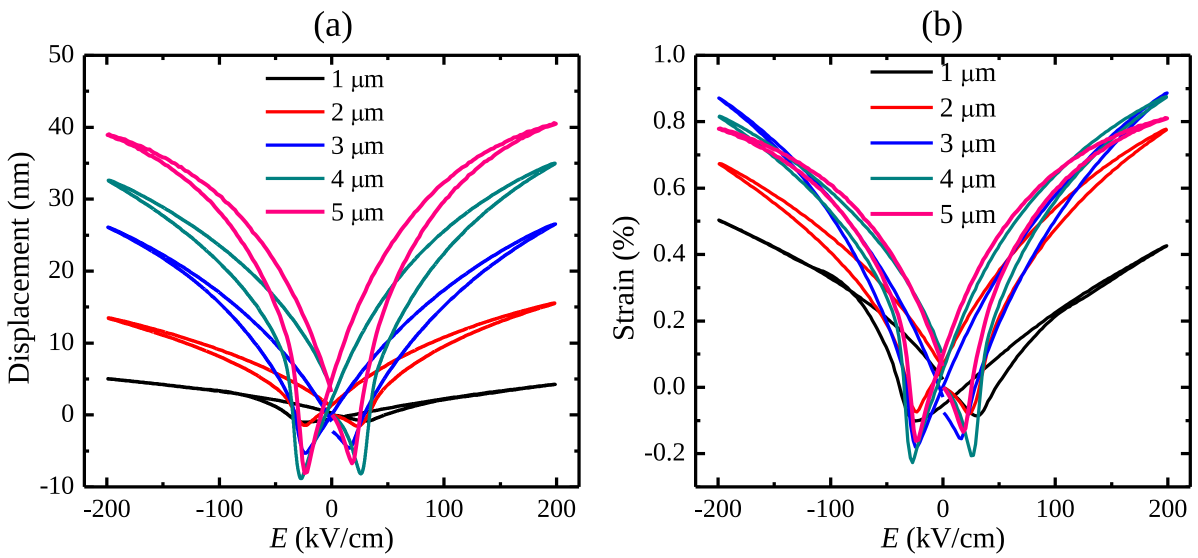

Figure S6. Large-signal bipolar (a) piezoelectric displacement versus *E*-field (*D*-*E*) and (b) piezoelectric strain versus *E*-field (*S*-*E*) loops of PZT films with different film thicknesses deposited on LNO/CNO*ns*/glass.

Figure S6a and b presents the typical butterfly-shaped piezoelectric displacement and piezoelectric strain curves of PZT films with various thicknesses, driven at increasing *ac* electric fields. The butterfly-like appearance of the displacement loops is attributed to the nature of domain motion and the piezoelectric properties of the PZT materials.

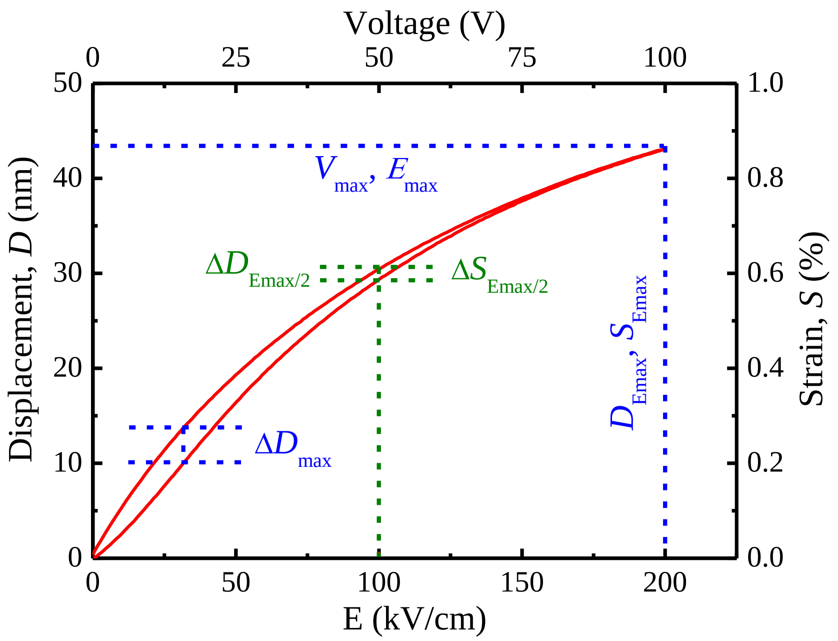

Figure S7. Piezoelectric strain hysteresis loop of 5-μm-thick PZT film deposited on LNO/CNO*ns*/glass.

From the unipolar piezoelectric displacement (strain) – electric field (voltage) loop, as shown in Fig. S7, the large-field piezoelectric strain coefficient (d_{33}^{*}), as an important parameter for actuator application, and the strain hysteresis (*H*), related to the piezoelectric loss, can be calculated as:

$$S(\%) = \frac{D}{t} \times 100 \tag{S1}$$

$$d_{33}^{*} = \frac{D_{Emax}}{V_{max}} = \frac{S_{Emax}}{E_{max}} \tag{S2}$$

$$H(\%) = \frac{\Delta S_{Emax/2}}{S_{Emax}} \times 100 = \frac{\Delta D_{Emax/2}}{D_{Emax}} \times 100 \tag{S3}$$

where, *t* is the thickness of PZT film.
